# Clinical significance of keratinocyte growth factor and K-sam gene expression in gastric cancer

**DOI:** 10.3892/mmr.2013.1397

**Published:** 2013-03-27

**Authors:** HIDEKI TANI, NOBORU SAITO, MAKIO KOBAYASHI, SHINGO KAMEOKA

**Affiliations:** 1 Department of Surgery II, Tokyo Women’s Medical University, Shinjuku-ku, Tokyo 162-8666, Japan; 2 Department of Pathology, Tokyo Women’s Medical University, Shinjuku-ku, Tokyo 162-8666, Japan

**Keywords:** keratinocyte growth factor, K-sam, gastric carcinoma, prognosis, risk factor, tumor marker

## Abstract

Although gastric cancer is increasingly being detected at an early stage of development, diffuse growth-type malignant tumors, such as scirrhous gastric cancer, are usually at an advanced stage at the time of diagnosis, resulting in poor treatment outcomes. The aim of this study was to determine whether the K-sam gene and keratinocyte growth factor (KGF) expression may be used to identify malignant tumors with a poor prognosis. K-sam and KGF expression was retrospectively evaluated in samples from 86 patients with early and advanced gastric cancer according to type, by examining serum levels and using immunohistochemical staining. The associations with clinicopathological characteristics and survival were also examined. The mean serum KGF levels were 11.191±3.808 pg/ml in early stage and 10.715±3.4991 pg/ml in advanced gastric cancer patients. KGF levels were significantly higher in types 4 and 5 (14.498±3.812 pg/ml, n=6) compared with types 1, 2 and 3 (10.747±3.571 pg/ml, n=80; P=0.028). Stage classification was identified as the only significant factor which determined overall survival. Patients with KGF-positive tumors had significantly higher serum KGF levels compared with those who had KGF-negative tumors. Patients with K-sam-positive tumors had significantly higher KGF levels compared with those who had K-sam-negative tumors. Pathological KGF expression was not significantly correlated with the degree of differentiation; however, there was a positive correlation between high K-sam expression in scirrhous gastric tumors and serum KGF levels. The present study revealed that high serum KGF levels are a risk factor for diffuse infiltrative gastric cancer and may provide a simple method of identifying patients with a poor prognosis among previously diagnosed preoperative gastric cancer patients.

## Introduction

Recent advances in diagnostic techniques have allowed gastric cancer to be detected early in an increasing number of cases and the treatment outcomes of gastric cancer as a whole have been significantly improved. However, diffuse growth-type malignant tumors, such as scirrhous gastric cancer, remain difficult to diagnose during the early stages of development and are usually at an advanced stage at the time of diagnosis, leading to poor treatment outcomes.

Cell motility is an important factor in primary tumor isolation and invasion; the cell adhesion factors that have been identified to promote cell motility include epidermal growth factor (EGF), transforming growth factor (TGF)-β and hepatocyte growth factor (HGF) (1–7). The present study investigated fibroblast growth factor (FGF), which is involved in fibroblast proliferation (8–11). With regard to the signal transmission pathways involved in scirrhous gastric cancer, scirrhous gastric cancer cells grow in the submucosa (SM), which is dense with lymph nodes, rather than on the mucosal surface where they would be exposed to gastric acid (12). FGF7 is produced by fibroblasts inside the stomach and promotes proliferation of scirrhous gastric cancer cells via FGFR2 receptors; the production of HGF and TGF-β promotes infiltration in a similar manner. Since TGF-β also promotes the proliferation of fibroblasts, these factors have the potential to induce the rapid infiltration and proliferation of scirrhous gastric cancer cells (13–15).

The K-sam gene, which is known to exacerbate scirrhous gastric cancer, has also been revealed to be homologous to other genes, including FGFR2, KGFR and Bek. An evaluation of the association of K-sam with keratinocyte growth factor (KGF) expression may enable the identification of malignant tumors with a poor prognosis (16–20).

## Materials and methods

### Patients and sample collection

A total of 86 patients underwent gastrectomy for carcinoma (early cancer, 49 cases; advanced cancer, 37 cases) between 1999 and 2003. The clinical characteristics of the patients are listed in Table I. A total of 31 patients who underwent surgery for a benign disease (e.g., inguinal hernia, gallbladder stone) and did not have cancer, as confirmed on prior screening, were enrolled as the control group in this study. Blood samples were obtained preoperatively from the 86 gastric carcinoma and 31 control patients. These samples were centrifuged and then preserved at −80°C in a freezer. The serum KGF concentration was estimated using a KGF ELISA kit and a clinical/oncological analysis was completed >5 years after the surgery.

A total of 86 patients who had undergone a primary tumor resection and were histologically diagnosed with sporadic gastric cancer were included in this study. None of the patients had received preoperative radiation therapy and/or chemotherapy. The samples were fixed in 10% formaldehyde solution and embedded in paraffin. The samples were sectioned (4-μm) and mounted on glass slides. Pathological diagnosis and classifications were determined according to the Japanese Classification of Gastric Carcinoma by the Japanese Gastric Cancer Association (21). Additionally, based on this classification, tumor invasion in the mucosa (M) and SM was determined as early-stage cancer, while tumor invasion in the muscularis propria (MP), subserosa (SS), serosa (SE), and adjacent structures (SI) was evaluated as advanced-stage cancer, regardless of lymph node or distant metastases.

### Antibodies and reagents

A rabbit polyclonal antibody against K-sam was purchased from Immuno-Biological Laboratories Co., Ltd. (9G-915, dilution 1:40; Gunma, Japan). A mouse monoclonal antibody against KGF was obtained from R&D Systems, Inc. (MAB2511, dilution 1:50; Minneapolis, MN, USA). Skim milk, Dako EnVision + System-HRP (DAB; catalog nos. K4006 and K4011), Target Retrieval Solution pH 9.0 and diaminobenzidine were purchased from Dako (Carpinteria, CA, USA).

Amplifying Wash Buffer™ 20× (catalog no. AA4; ProHisto, Columbia, SC, USA), xylene, ethanol, H_2_O_2_, coverslips (24×50 mm, no. 1 thickness; Chase Scientific Glass, Inc., Rockwood, TN, USA) and hematoxylin (Gill-1, catalog no. 23-245653; Thermo Fisher Scientific, Inc., Kanagawa, Japan) were also used in this study.

### Serological investigation

The venous blood samples, which were obtained prior to surgery, were centrifuged and the purified serum was stored at −80°C. The frozen serum was allowed to thaw naturally prior to examination. Serum KGF levels were measured using the ELISA method with a Quantikine Human KGF ELISA kit. A cuvette port internal microplate spectrophotometer was used, with a biquadratic approximation formula.

Subsequently, 100 μl assay diluent and 100 μl of sample were added to each well. The wells were covered with the adhesive strip provided and then incubated for 3 h at room temperature. Each well was aspirated and washed 4 times; for washing, each well was filled with wash buffer (400 μl).

KGF conjugate (200 μl) was added to each well. The wells were covered with a new adhesive strip and incubated for 2 h at room temperature. Each well was then aspirated and washed 4 times, as previously described. Substrate solution (200 μl) was then added to each well, followed by incubation.

### Immunohistochemical techniques

The immunohistochemical detection of K-sam and KGF was performed according to the manufacturer’s instructions. Briefly, the slides were deparaffinized in xylene and hydrated in decreasing concentrations of ethyl alcohol. The tissues were heated for 20 min at 105°C by autoclave in Target Retrieval Solution pH 9.0. Then, the sections were deparaffinized and incubated with 3% hydrogen peroxide in methanol for 15 min to block the endogenous peroxidase activity. The sections were washed in phosphate-buffered saline (PBS) and incubated in skim milk for 10 min to reduce the non-specific antibody binding.

The specimens were incubated with K-sam (2.5 mg/ml) or KGF antibody (2.5 mg/ml) overnight at 4°C, followed by 3 washes with PBS. The sections were incubated with labeled polymer-HRP (bottle 2) for 30 min at room temperature, followed by 3 washes with PBS. The slides were treated with ready-to-use AEC substrate-chromogen solution for 3 min and washed with PBS 3 times. Finally, the slides were incubated in PBS diaminobenzidine and 1% hydrogen peroxide v/v for 60 or 90 sec, counterstained with Mayer’s hematoxylin and mounted.

### Identification of K-sam and KGF using immunohistochemical staining

Known cases of scirrhous gastric cancer were used as controls for KGF and K-sam expression. KGF was immunologically localized mainly in the cytoplasm, while K-sam was in the cell membrane and cytoplasm (22). Hematoxylin and eosin (H&E) staining was also used on the control slides to select infiltrative regions. Infiltrative regions were defined as areas on the serosal side where cancer cells were initially observed. Microscopy revealed that cancer cells were continuously present from the mucosa to the serosa.

**Figure 1:**
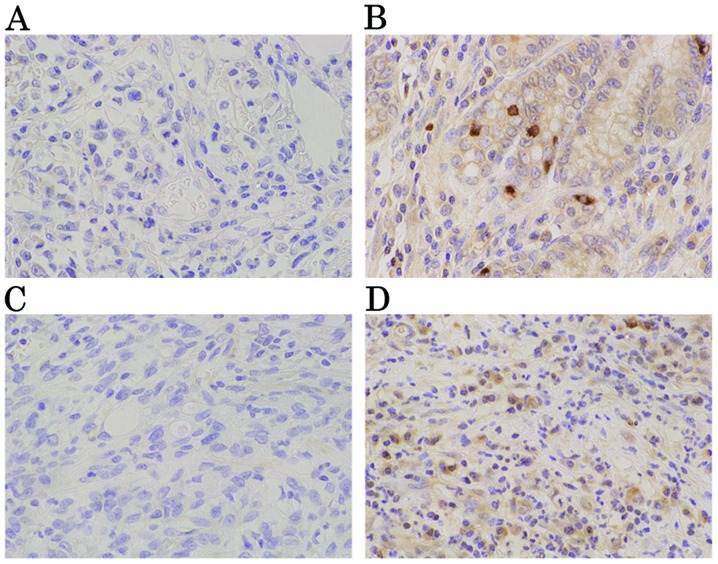
Immunohistochemical determination of K-sam and KGF. KGF, keratinocyte growth factor. (A and B) Tumors were evaluated as positive for K-sam when ≥50% of tumor cells in the infiltrative region were stained more intensely than healthy epithelial cells in the same region. (C and D) In the infiltrative region, tumors were evaluated as positive for KGF when ≥2 fibroblasts in the interstitium were stained more intensely than the fundic gland (magnification, ×200).

K-sam antibodies were weakly stained in the gastric cancer mucosa. Tumors were evaluated as positive for K-sam when ≥50% of tumor cells in the infiltrative region were stained more intensely than healthy epithelial cells in the same region (Fig. 1A and B). In the infiltrative region, tumors were evaluated as positive for KGF when ≥2 fibroblasts in the interstitium were stained more intensely than the fundic gland at a magnification of ×200 (Fig. 1C and D).

### Statistical analysis

The Chi-square and Mann-Whitney U tests were used to determine the significance of the differences between the covariates. The survival durations were calculated using the Kaplan-Meier method and analyzed using the log-rank test to compare the cumulative survival durations in the patient groups. Additionally, a Cox proportional hazards model was used to determine multivariate hazard ratios for the study parameters. In all the tests, P<0.05 was considered to indicate a statistically significant difference. The JMP 8.0.1 software program (SAS Institute, Inc., Cary, NC, USA) was used for the analysis.

## Results

### Association of serological KGF levels with clinicopathological factors

**Figure 2:**
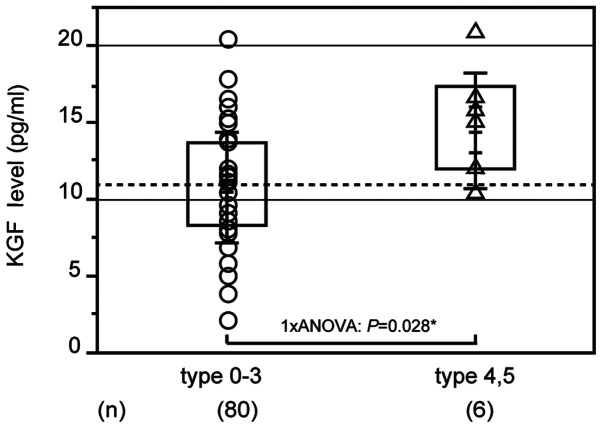
Association between serum KGF levels and the macroscopic type. KGF, keratinocyte growth factor.

**Figure 3:**
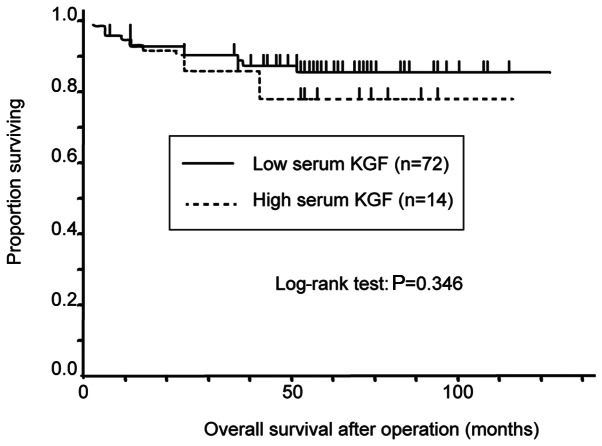
Overall survival using the Kaplan-Meier method in the groups classified as high- or low-KGF, with a cut-off value of 14.608 pg/ml (the mean + 1xSD). The high-KGF group exhibited a tendency towards a poorer prognosis. KGF, keratinocyte growth factor.

Fig. 2 shows the association between clinical/oncological factors and serum KGF levels. The average KGF level in early-stage gastric cancer was 11.191±3.808 pg/ml and in advanced gastric cancer was 10.715±3.4991 pg/ml; the difference between these results was small. Analysis of the macroscopic type revealed that KGF levels were significantly higher in types 4 and 5 (14.498±3.812 pg/ml, n=6) compared with types 1, 2 and 3 (10.747±3.571 pg/ml, n=80; P=0.028; Fig. 2). With regard to the serum KGF levels, there were no significant differences in histological type, invasion depth, lymph node infiltration, vascular infiltration, lymph node metastasis or stage classification (Table II). For the overall survival rate, stage classification was the only significant factor that determined prognosis (Table III). Tumors were classified as high- or low-KGF with a cut-off value of 14.608 pg/ml (the mean + 1xSD). The Kaplan-Meier method was used to calculate the survival rate curves, with the high-KGF group trending towards a poorer prognosis (Fig. 3).

### Pathological KGF/K-sam expression

For the association between pathological KGF expression and histological type, there was a tendency for KGF-positive tumors to be poorly differentiated; however, this difference was not significant.

**Figure 4:**
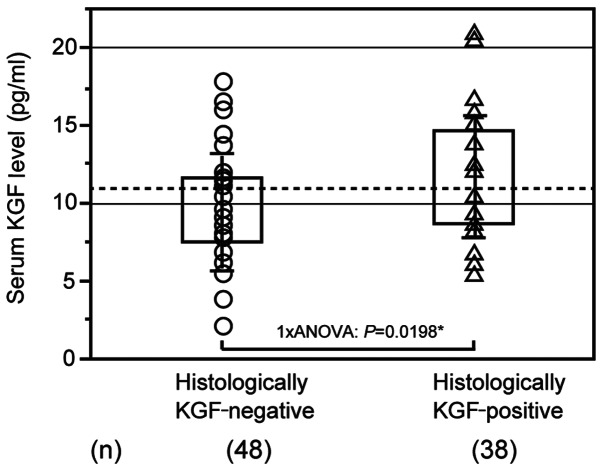
Association between serum KGF levels and the histological presence of KGF. KGF, keratinocyte growth factor.

Exploring the association between pathological KGF expression and serum KGF levels revealed that the mean level for patients with KGF-negative tumors was 10.121±3.329 pg/ml, while it was 12.131±3.861 pg/ml for patients with KGF-positive tumors. Patients with KGF-positive tumors had significantly higher KGF levels compared with patients with KGF-negative tumors (Fig. 4).

**Figure 5:**
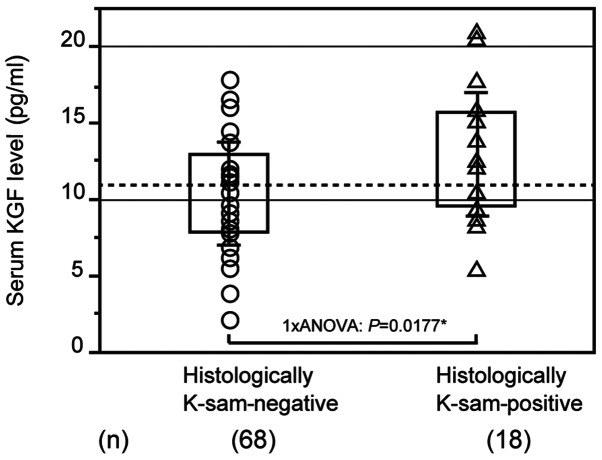
Association between serum KGF levels and the histological presence of K-sam. KGF, keratinocyte growth factor.

As demonstrated in previous studies, the K-sam gene is highly expressed in scirrhous gastric cancer (22). The mean KGF level was 10.456±3.362 pg/ml in patients with K-sam-negative tumors and 13.099±4.212 pg/ml in patients with K-sam-positive tumors. Patients with K-sam-positive tumors had significantly higher KGF levels than those with K-sam-negative tumors (Fig. 5).

Pathological KGF expression was not significantly correlated with the degree of differentiation, while there was a positive correlation between high K-sam expression and serum KGF levels in scirrhous gastric tumors.

Patients with a high expression of the K-sam gene also tended to have high serum KGF levels. It is possible that serum KGF levels may be correlated with high K-sam expression and diffuse infiltrative gastric cancer.

## Discussion

Various mechanisms have been hypothesized to exist in the interactions between cancer and interstitial cells. Interstitial cell growth and angiogenesis factors produced by cancer cells are considered to trigger interstitial cell recruitment and angiogenesis. It has also been shown that cells recruited by cancer cells produce various tumor growth factors which promote growth and give rise to the invasive capacity of cancer cells. In a number of these molecular mechanisms, the surrounding cells are hypothesized to have paracrine-like functions, resulting from the molecules produced and released by cancer or interstitial cells (15).

The growth factors previously investigated, including KGF and K-samII, have been shown to mainly contribute to fibrosis. The molecules involved in this process have an impact on cancer cells, fibroblasts and inflammatory cells, creating the properties required for rapidly progressing, diffuse infiltrative gastric cancer cells (13).

In the present study, the mechanisms that control the development of diffuse infiltrative gastric cancer were examined through the serological and pathological evaluation of fibroblast growth factors and their receptors and coding genes. Serum KGF levels in patients with gastric cancer were demonstrated to be higher in those who had tumors with a poor prognosis and exhibited significant cell proliferation and infiltration. This suggests that the K-sam gene, which codes for the pathological KGF receptor KGFR, was highly expressed. Therefore, it is possible that KGFR expression may also be high and that KGF is released into the serum, leading to the association between the high expression of the K-sam gene and serum KGF levels.

Serum KGF was significantly higher in patients with macroscopic types 4 and 5, regardless of the stage of cancer progression. This finding indicates that it may be possible to search serologically for malignant tumors with a poor prognosis that exhibit significant proliferation and infiltration, such as scirrhous gastric cancer.

Previous studies have shown that the types of gastric cancer that have a high pathological expression of K-sam or KGF are malignant tumors with a poor prognosis that induce significant proliferation and infiltration, such as scirrhous gastric cancer. Therefore, this has been identified as one factor which may determine prognosis (23,24).

In the present study, there was a positive correlation between pathological KGF expression and the serum KGF levels, in addition to a positive correlation between pathological K-sam expression and KGF levels, which indicates an association between serum KGF and diffuse malignant tumors. Since there was no correlation with cancer invasion depth or the stage of progression, it may be possible to identify gastric cancer patients who have the potential to develop malignant tumors with a poor prognosis that exhibit significant proliferation and infiltration, such as scirrhous gastric cancer, at the precursor lesion stage. However, the investigation of survival rates conducted in this study did not identify serum KGF as a factor determining prognosis. Further studies are required to elucidate its clinical significance as a biomarker.

Currently, diffuse growth-type malignant tumors, such as scirrhous gastric cancer, remain difficult to diagnose during the early stages of development and are usually at an advanced stage at the time of diagnosis, which leads to poor treatment outcomes.

The results of the present study suggest that serum KGF is a risk factor for diffuse infiltrative gastric cancer and may provide a simple method of identifying patients with a poor prognosis among previously diagnosed preoperative gastric cancer patients.

## Figures and Tables

**Table I:** Patient characteristics.

Characteristics	n (%)
Age, mean (SD)	63.33 (9.5)
Gender	
Male	66 (76.74)
Female	20 (23.26)
Macroscopic types	
Type 0	53 (61.63)
Type 1	4 (4.65)
Type 2	15 (17.44)
Type 3	8 (9.30)
Type 4	5 (5.81)
Type 5	1 (1.16)
Histological types	
Papillary	2 (2.33)
Well-differentiated (tub1)	27 (31.40)
Moderately differentiated (tub2)	16 (18.60)
Poorly differentiated (por1)	20 (23.26)
Poorly differentiated (por2)	12 (13.95)
Signet-ring cell	6 (6.98)
Mucinous	3 (3.49)
Depth of tumor invasion	
M	22 (25.58)
SM	27 (31.40)
MP	8 (9.30)
SS	13 (15.12)
SE	16 (18.61)
Lymphatic invasion	
ly0	37 (43.02)
ly1	31 (36.05)
ly2	14 (16.28)
ly3	4 (4.65)
Venous invasion	
v0	69 (80.23)
v1	16 (18.61)
v2	1 (1.16)
Lymph node metastasis	
n0	63 (73.26)
n1	14 (16.28)
n2	6 (6.98)
n3	1 (1.16)
n4	2 (2.33)
Japanese clinical stage	
Ia	46 (53.49)
Ib	14 (16.28)
II	12 (13.95)
IIIa	5 (5.81)
IIIb	3 (3.49)
IVa	2 (2.33)
IVb	4 (4.65)

SD, standard deviation; M, mucosa; SM, submucosa; MP, muscularis propria; SS, subserosa; SE, serosa.

**Table II:** Univariate analysis of serum KGF.

Factors	n	Mean	SD	P-value
Histological types				
Papillary/well- to moderately differentiated	45	11.242	3.797	NS
Poorly differentiated/signet-ring cell/mucinous	41	10.753	3.601	
Depth of tumor invasion				
M/SM	49	11.191	3.808	NS
MP/SS/SE	37	10.768	3.568	
Lymphatic invasion				
ly0/ly1	68	10.949	3.72	NS
ly2/ly3	18	11.235	3.68	
Venous invasion				
v0	69	10.758	3.445	NS
v1/v2	17	12.029	4.537	
Lymph node metastasis				
No metastasis	64	11.253	3.594	NS
Metastasis	22	10.3	3.963	
Japanese clinical stage				
Ia/Ib/II	72	11.015	3.573	NS
IIIa/IIIb/IVa/IVb	14	10.977	4.403	
Macroscopic types				
Type 0/1/2/3	80	10.747	3.571	P=0.028
Type 4/5	6	14.498	3.812	

KGF, keratinocyte growth factor; SD, standard deviation; NS, not significant; M, mucosa; SM, submucosa; MP, muscularis propria; SS, subserosa; SE, serosa.

**Table III:** Univariate and multivariate analysis of overall survival.

	Multivariate		Univariate		
			
Factors	Risk ratio	95% CI	Risk ratio	95% CI	P-value
Depth of tumor invasion (M, SM) vs. (MP, SS, SE)	1.159	0.033–35.213	16.778	3.337–304.762	NS
Infiltration (α, β) vs. (γ)	1.468	0.049–43.966	18.568	3.692–377.311	NS
Vascular invasion (v0) vs. (v1, v2)	5.133	0.728–34.157	8.614	2.858–28.649	NS
Lymphatic invasion (ly0, ly1) vs. (ly2, ly3)	0.329	0.054–1.757	7.278	2.474–23.928	NS
Histological type (Papillary/well- to moderately differentiated) vs. (poorly differentiated/signet-ring cell/mucinous)	2.158	0.478–15.611	6.389	1.714–41.301	NS
Lymph node metastasis (No metastasis) vs. (metastasis)	0.532	0.040–6.389	0.053	0.008–0.197	NS
Japanese clinical stage (Ia, Ib, II) vs. (IIIa, IIIb, IVa, IVb)	28.004	2.770–937.265	48.436	12.788–315.979	0.00031
Macroscopic type (Type 0, 1, 2, 3) vs. (type 4, 5)	0.714	0.092–6.209	20.306	6.193–66.794	NS
Serum KGF level (≥mean + 1xSD) vs. (<mean + 1xSD)	1.124	0.225–5.807	1.731	0.462–5.300	NS

CI, confidence interval; M, mucosa; SM, submucosa; MP, muscularis propria; SS, subserosa; SE, serosa; NS, not significant; KGF, keratinocyte growth factor.
